# Sepsis-induced Autoimmune Hemolytic Anemia: A Case Report

**DOI:** 10.5811/cpcem.2020.8.49040

**Published:** 2020-10-05

**Authors:** Zach Edwards, Stephen DeMeo

**Affiliations:** * Campbell University School of Osteopathic Medicine, Raleigh, North Carolina; † WakeMed, Department of Neonatology, Raleigh, North Carolina

**Keywords:** Autoimmune hemolytic anemia, sepsis, sepsis-induced autoimmune hemolytic anemia

## Abstract

**Introduction:**

Sepsis commonly brings patients to the emergency department (ED). Patient outcomes can vary widely. In some cases, rare complications of sepsis such as autoimmune hemolytic anemia can occur.

**Case Report:**

A 68-year-old female presented with sepsis secondary to infected nephrolithiasis. The patient had signs and symptoms consistent with hemolysis upon arrival to the ED. Her hemolysis progressively worsened over a two-day period leading to a diagnosis of warm autoimmune hemolytic anemia. She responded well to treatment; however, her condition began to worsen due to a new infection caused by perforated colonic diverticula. The patient ultimately expired from complications of her perforated colonic diverticula.

**Conclusion:**

It is crucial that emergency physicians understand the risk factors, symptoms, pathophysiology, and treatment of this rare complication of sepsis so that favorable patient outcomes can be achieved.

## INTRODUCTION

Globally, it is estimated that between 20–30 million people develop sepsis annually and that eight million will die each year.^1^ Prompt treatment and identification of the infectious etiology is key in preventing end-organ damage and or death. In rare instances, patients can develop complications from sepsis; one such complication is autoimmune hemolytic anemia. This patient’s complicated hospital course offers valuable insight on sepsis-induced autoimmune hemolytic anemia.

## CASE REPORT

A 68-year-old female with a past medical history of atrial fibrillation, deep vein thrombosis, pulmonary embolism, nephrolithiasis, diverticulitis, and asthma presented with two days of flank pain, abdominal pain, subjective fever, nausea, vomiting, and diarrhea. On physical exam the patient was noted to have bilateral costovertebral angle tenderness, mild diffuse abdominal pain, scleral icterus, and a negative Murphy’s sign. Her blood pressure was 80/64 millimeters of mercury, heart rate 116 beats per minute, and temperature 97.9 degrees Fahrenheit.

Initial laboratory values demonstrated a leukocyte count of 26,400 microliters (μL) (reference [ref] range 4000–10,000/μL); hemoglobin 9.7 milligrams per deciliter (mg/dL) (ref range 11.4 – 15 mg/dL); total bilirubin 8.5 mg/dL (ref range 0.3 – 1.0 mg/dL); direct bilirubin 1.5 mg/dL (ref range 0.0 – 0.2 mg/dL); lactic acid 6.6 millimoles per liter (mmol/L) (ref range 0.67–1.8 mmol/L); and creatinine 2.37 mg/dL (ref range 0.7–1.3 mg/dL). The patient’s urine was positive for nitrites (ref negative result), leukocytes (negative), and blood (negative). A non-contrast abdominal computed tomography demonstrated a left renal pelvic stone and evidence of sigmoid diverticulosis without diverticulitis. Broad-spectrum antibiotics, fluids, and vasopressors were started immediately. The patient was started on intravenous (IV) ceftriaxone, metronidazole, and vancomycin. Five hours after her arrival she was switched to cefepime and vancomycin.

Urology was consulted and opted to emergently place a left renal stent to alleviate the obstruction. On the second day, her hemoglobin decreased to 5.8 grams (g)/dL (ref range 12–16 g/dL) requiring a blood transfusion with two units of packed red blood cells. Prior to transfusion, she was found to have a positive direct Coombs test and spherocytes on the peripheral blood smear. There were no schistocytes on her peripheral blood smear (which can be seen in microangiopathic hemolytic anemia and disseminated intravascular coagulation). She was diagnosed with warm autoimmune hemolytic anemia secondary to sepsis. The patient’s key lab values at the time of diagnosis can be seen in Table. Cephalosporin-induced hemolytic anemia was considered; however, the patient was hemolyzing prior to presenting to the ED, which made sepsis a more likely culprit. The patient was started on IV methylprednisolone 60 mg every six hours. Over the next 24 hours the patient’s signs and symptoms of hemolysis began to improve.

On the eighth day of the patient’s hospital course her autoimmune hemolytic anemia began to worsen due to a new infectious process. Her condition deteriorated rapidly, which lead to septic shock. An abdominal computed tomography was performed and demonstrated pneumoperitoneum, abscess, and viscous perforation. She underwent an exploratory laparotomy, which revealed peritonitis due to several perforated colonic diverticula, and resulted in a hemicolectomy and sigmoidectomy. Shortly after being transferred to the intensive care unit postoperatively, the patient underwent cardiac arrest. The healthcare team was unable to obtain the return of spontaneous circulation and the patient expired.

## DISCUSSION

Autoimmune hemolytic anemia is a rare blood cell disorder with an incidence of 1–3 per 100,000 people per year.^2^ This disorder can be broken down into primary (idiopathic) and secondary (due to a known trigger).^3^ Furthermore, there are two distinct types, cold agglutinin-mediated autoimmune hemolytic anemia and warm autoimmune hemolytic anemia.^4^ Warm autoimmune hemolytic anemia occurs far more often than the cold variant.^5^ Between 70–80% of autoimmune hemolytic anemia cases in adults are the warm variant.^2^,^6^ This blood cell disorder has several etiologies. These include infection, malignancy (chronic leukocytic leukemia), autoimmune disorders (systemic lupus erythematosus), and many medications (most notably cephalosporins).^2^,^4^,^7^,^8^

CPC-EM Capsule

What do we already know about this clinical entity?

*Autoimmune hemolytic anemia is a rare complication of sepsis*.

What makes this presentation of disease reportable?

*There are few published case reports on sepsis-induced autoimmune hemolytic anemia*.

What is the major learning point?

*This case highlights the epidemiology, pathophysiology, signs, symptoms, and treatment for autoimmune hemolytic anemia*.

How might this improve emergency medicine practice?

*Understanding this rare complication of sepsis will help providers diagnose and treat the disease*.

Warm autoimmune hemolytic anemia is an example of a type II hypersensitivity reaction. The pathophysiology behind this disorder involves immunoglobulin G antibodies attacking red blood cells, resulting in extravascular hemolysis.^8^ These immunoglobulins react with red blood cells at temperatures near 37ºC.^3^ This causes red blood cells to be coated with antibodies that signal the immune system to destroy the red blood cells.^2^ The host’s red blood cells are destroyed in an antibody-dependent manner that is carried out by cytotoxic CD8+T cells and natural killer cells within the spleen.^2^ Splenic macrophages also play a significant role in the extravascular hemolysis of the host’s red blood cells, primarily through phagocytosis.^5^ The complement system contributes to a lesser degree in warm autoimmune hemolytic anemia vs cold agglutinin disease.^2^,^6^

Patients typically present with signs and symptoms of anemia and hemolysis. Symptoms include pallor, jaundice, splenomegaly, and dark urine.^3^,^5^,^8^ These symptoms are not specific and can present in a variety of disorders. However, if the clinical picture aligns with this disorder (eg, recent cephalosporin use, infection, malignancy) and these symptoms present, then warm autoimmune hemolytic anemia should be on the clinician’s differential diagnosis. Laboratory findings include reduced hemoglobin, hematocrit, and haptoglobin. Patients will also have elevated lactate dehydrogenase and indirect bilirubin.^5^,^6^,^7^ The peripheral blood smear will demonstrate spherocytosis.^5^ The presence of hemolytic anemia, positive direct Coombs test, and spherocytes are required to make a diagnosis of autoimmune hemolytic anemia.^5^,^7^

Initial management of patients with this disorder should be to determine whether a blood transfusion is necessary. This decision should be guided by the hemoglobin level, symptoms, and risk factors of the patient. Hemoglobin levels less than 7 mg/dL usually require a blood transfusion. Clinicians should also assess the need for venous thromboembolism prophylaxis, as one study found between 15–33% of patients diagnosed with warm autoimmune hemolytic anemia experienced venous thromboembolism.^9^ The first-line treatment for this disorder is with oral prednisone or intravenous (IV) high-dose steroids such as methylprednisolone.^2^ If the patient does not adequately respond to steroids, then other treatments such as IV immunoglobulin or plasmapheresis may be used. Second-line therapy includes rituximab and splenectomy.^2^,^6^ Mortality rates for autoimmune hemolytic anemia are low and are usually related to infection secondary to splenectomy.^7^ Although most patients achieve full remission some will have a chronic relapsing course.^5^

## CONCLUSION

Nephrolithiasis may induce septic shock, which can result in autoimmune hemolytic anemia. Knowing the symptoms and risk factors for this disorder will allow emergency physicians to add it to their differential when appropriate. To increase favorable patient outcomes, emergency physicians must understand the treatment and pathophysiology of warm autoimmune hemolytic anemia.

## Figures and Tables

**Table:** Patient’s laboratory data upon diagnosis of warm autoimmune hemolytic anemia.

Variable	Reference range	Patient’s labs upon diagnosis
Hemoglobin	11.4–15 mg/dL	5.8 mg/dL
Hematocrit	31–42%	17%
Platelet	150 – 450 K/μL	204
Total bilirubin	0.3–1.0 mg/dL	33.5 mg/dL
Direct bilirubin	0.0–0.2 mg/dL	20.6 md/dL
Haptoglobin	44–215 mg/dL	<30 mg/dL
Lactate dehydrogenase	140–271 IU/L	657 IU/L

*mg*, milligram; *dL*, deciliter; *μL*, microliter; *K*, thousand; *IU*, international unit; *L*, liter.
